# Evaluation of early atherosclerotic findings in women with polycystic ovary syndrome

**DOI:** 10.1186/1757-2215-4-19

**Published:** 2011-10-24

**Authors:** Afshin Mohammadi, Mohammadreza Aghasi, Leila Jodeiry-farshbaf, Shaker Salary-Lac, Mohammad Ghasemi-rad

**Affiliations:** 1 Department of Radiology, Urmia University of Medical Sciences, Urmia, Iran; 2 Department of Endocrinology, Urmia University of Medical Sciences, Urmia, Iran; 3 Department of Internal Medicine, Urmia University of Medical Sciences, Urmia, Iran; 4 Department of public health, Urmia University of Medical Sciences, Urmia, Iran; 5 Student research committee, Urmia University of Medical Sciences, Urmia, Iran

## Abstract

**Background**

Polycystic ovary syndrome (PCOS) is the most common endocrinopathy in women of childbearing age, and it seems better to consider it as an ovarian manifestation of metabolic syndrome. The aim of the current study was to evaluate early atherosclerotic findings in patients with PCOS.

**Methods**

We enrolled 46 women with PCOS and 45 normal control subjects who were referred to our hospital's endocrinology outpatient clinic. Carotid intima media thickness (CIMT) and flow-mediated dilatation (FMD) were performed in both cases and matched controls.

**Results**

Patients with PCOS showed an increased mean CIMT (0.63 ± 0.16 mm) when compared with the control subjects (0.33 ± 0.06 mm). This difference was statistically significant (p = 0.001). The mean FMD in young patients with PCOS was 10.07 ± 1.2%, while it was 6.5 ± 2.06% in normal subjects. This difference was also statistically significant (p = 0.001).

**Conclusion**

Our findings suggest that PCOS is related with early atherosclerotic findings.

## Background

Polycystic ovary syndrome (PCOS) is the most common endocrinopathy in women of childbearing age, and it seems better to consider it as an ovarian manifestation of metabolic syndrome (MS) [1,2]. MS has already been confirmed as part of the **tsunami **of cardiovascular risk factors (obesity, lipid abnormalities, impaired glucose tolerance and hypertension) [3]. Insulin resistance is considered as the basic pathophysiological mechanism in MS, and is also a well-recognised presentation of PCOS [4]. However, data regarding endothelial function impairment as an indicator of increased risk of cardiovascular disease in **PCOS **are still controversial [5,6], with some studies saying that PCOS-induced insulin resistance does not show endothelial dysfunction [7]. The aim of our study was to assess and compare the endothelial function as a predictor of cardiovascular risk by measuring flow-mediated dilatation in young women with PCOS and matched control subjects.

## Method

Before the beginning of the study, its protocol was approved by the University Ethics Committee and the Faculty of Medicine. Written informed consent was obtained from each participant. We enrolled 46 women with PCOS and 45 normal control subjects who were referred to our hospital's endocrinology outpatient clinic. The patients and controls were selected from the normotensive population with a body mass index (BMI) less than 27 kg/m^**2**^. Women with diabetes mellitus, cases of hypertension and those with age above 30 years and BMI above 27 kg/m^2 ^were excluded from study.

The diagnosis of PCOS was documented based on a history of oligomenorrhoea or amenorrhoea (less than eight cycles per year), clinical or biochemical manifestations of hyperandrogenism such as hirsutism, acne or elevation of at least one circulating ovarian androgen (serum dehydroepiandrosterone sulphate [DHEAS] or testosterone) and polycystic ovaries at ultrasound. Two of three criteria were sufficient to confirm the diagnosis. Controls were healthy women with normal menstrual cycles, non-hyperandrogenic, non-hirsute and with normal ovarian morphology at ultrasound. One examiner (M.A.) assessed the hirsutism according to Ferriman-Gallwey score; a score greater than 7 was considered to indicate hirsutism.

All those with secondary causes of hyperandrogenism, such as hyperprolactinaemia, thyroid disease, late onset congenital adrenal hyperplasia (17-OH progesterone > 2 ng/dl), androgenic tumour (testosterone > 4 ng/ml), Cushing disease, renal or liver failure, pregnancy and diabetes mellitus, were excluded from the study. After basic history taking, anthropometric properties of cases and controls such as BMI, waist circumference (WC), hip circumference (HC), ratio of WC/HC and systolic and diastolic blood pressure were measured.

Fasting blood samples were collected for measurement of blood glucose, insulin, androgens, triglycerides (TG), total cholesterol (TC), high-density lipoprotein (HDL) and low-density lipoprotein (LDL). Insulin resistance (IR) was assessed using both fasting insulin levels and the homeostasis model assessment (HOMA) calculation: fasting serum insulin (micro units per millilitre) multiplied by fasting plasma glucose (millimoles per litre) and divided by 22.5.

The serum levels of DHEAS, follicle-stimulating hormone (FSH), luteinising hormone (LH), 17 hydroxy (OH) progesterone, testosterone and prolactin were also measured in cases and controls.

### CIMT measurement

High-resolution B mode ultrasonographies of both the common and internal carotid arteries were performed with an ultrasound device (Siemens, Sonoline G40, Germany) equipped with a 10 MHz linear array transducer. Patients were examined in the supine position with the head tilted backwards. After the carotid arteries were located by transverse scan, the probe was rotated 90° to obtain and record a longitudinal image of common carotid arteries.

The maximum CIMT was measured at the posterior wall of the common carotid artery, 2 cm before the bifurcation, as the distance between first and second echogenic lines of anterior and posterior arterial walls. The image was focused on the posterior wall of the common carotid artery, and we used the gain settings to optimise the quality of the image. For accuracy, the CIMT measurements were performed vertical to the arterial wall. Three CIMT measurements were taken at each site and the average measurement was calculated and used.

### Flow-mediated dilatation (FMD) measurement

Ultrasound examination of FMD was performed in the morning after overnight fast, after 15 minutes rest in the horizontal position, by means of a Sonoline G40 ultrasound scanner (Siemens, Germany) with a linear transducer (10 MHz). The diameter of the right brachial artery was measured 3-5 cm above the antecubital space at baseline. The measurement was performed in the end-diastolic phase, marking the diameter between anterior and posterior artery wall in the zone between the media and adventitia ('m-line'). An average of three measurements was taken and further analysed to calculate FMD. Subsequently, a pneumatic tourniquet was placed on the upper part of the right forearm and inflated for four minutes to a pressure of 200 mm Hg or 50 mm Hg above systemic arterial blood pressure. Sixty seconds after cuff release, the diameter of the right brachial artery was measured three times. FMD was calculated as an increase of vascular diameter (in percentage) from the difference between maximum and baseline brachial artery diameter. Data were calculated as absolute diameter of the brachial artery (in mm) and percentage increased in the diameter of the brachial artery. CIMT and FMD in all cases and controls were measured by one radiologist (A.M.), who was blinded to clinical and laboratory data of patients and controls.

Statistical analysis was performed using SPSS (version 16. Chicago, IL, USA). We performed the statistical calculation by using the T-test, Mann-Whitney U test, Kolmogorov-Smirnov (K-S) test and logistic regression. A p value equal or less than 0.05 was considered statistically significant.

## Results

The mean ± SD of age was 23.02 ± 5.17 in the patient group and 27.96 ± 3.97 in the control group. The mean ± SD of BMI in PCOS was 25.08 ± 5.54 kg/m^2^, and in control subjects it was 21.59 ± 3.08 kg/m^2^. There were statistically significant differences in age, BMI, AC, HC and ratio of AC/HC between cases and control subjects. Table 1 summarises the anthropometrics data of PCOS and control subjects.

**Table 1:** This table shows the anthropometric data of the cases and controls.

Variable	Group	Number	Mean ± SD	P value
Age	Control	45	27.96 ± 3.97	0.000
	PCOS	46	23.02 ± 5.17	
Height	Control	45	163.78 ± 3.80	0.03
	PCOS	46	161.43 ± 6.40	
Weight	Control	45	58.73 ± 7.44	0.008
	PCOS	46	65.60 ± 15.25	
BMI	Control	45	21.59 ± 3.80	0.001
	PCOS	46	25.80 ± 5.45	
AC	Control	45	80.16 ± 12.76	0.65
	PCOS	46	81.61 ± 12.86	
HC	Control	45	97.87 ± 6.20	0.003
	PCOS	46	105.96 ± 16.46	
WC/HC	Control	45	0.82 ± 0.4	0.02
	PCOS	46	0.79 ± 0.09	

The mean systolic blood pressure (SBP) and diastolic blood pressure (DBP) in PCOS patients and control subjects were 114.46 ± 29.02 mm/Hg, 79.02 ± 8.40 mm/Hg and 120.67 ± 18.26 mm/Hg, 78.40 ± 7.37 mm/Hg, respectively. There was no statistically significant difference between PCOS and controls in terms of SBP and DBP. There were statistically significant differences in terms of FBS, TG, TC, LDL and HDL between PCOS and control subjects. Table 2 summarises the SBP, DBP and biochemical data of PCOS patients and control subjects.

**Table 2:** This table shows the systolic and diastolic blood pressure and biochemical data of the cases and controls.

SBP (mm/Hg)	Controls	45	120.67 ± 18.26	0.22
	Cases	46	114.46 ± 29.02	
DBP (mm/Hg)	Controls	45	78.44 ± 7.37	0.72
	Cases	46	79.02 ± 8.40	
FBS (mg/dl)	Controls	45	74.73 ± 9.28	0.0001
	Cases	46	85 ± 8.67	
TG (mg/dl)	Controls	45	98.76 ± 48.90	0.04
	Cases	46	123.02 ± 62.70	
LDL (mg/dl)	Controls	45	118.79 ± 31.76	0.05
	Cases	46	133.43 ± 40.24	
HDL (mg/dl)	Controls	45	60.16 ± 13.16	0.0001
	Cases	46	47.49 ± 8.41	

Assessment of sex hormone and insulin levels between PCOS and control subjects showed that there was a significant difference in term of FSH, prolactin (PRL), testosterone and DHEAS between cases and controls, but there were no significant differences between cases and controls in term of LH and 17 OH progesterone levels. Table 3 summarises the serum insulin and sex hormone levels in PCOS patients and controls.

**Table 3:** This table shows the serum hormonal characteristics of the cases and controls.

Variable	Groups	Number	Mean ± SD	P Value
FSH (mIu/ml)	Controls	45	5.57 ± 1.88	0.01
	Cases	46	7.33 ± 4.25	
LH (mIu/ml)	Controls	45	5.46 ± 4.72	0.24
	Cases	46	26.51 ± 14.63	
PRL (ng/ml)	Controls	45	13.61 ± 5.39	0.001
	Cases	46	21.15 ± 19.11	
Testosterone	Controls	45	0.55 ± 0.87	0.001
	Cases	46	1.58 ± 1.21	
DHEAS (g/dl μ)	Controls	45	187.22 ± 105.61	0.001
	Cases	46	284.48 ± 111.18	
17(OH) Progesterone (ng/dl)	Controls	45	1.25 ± 0.76	0.23
	Cases	46	1.69 ± 2.35	
Insulin (mIu/ml)	Controls	45	4.34 ± 1.90	0.001
	Cases	46	12.62 ± 8.13	

Patients with PCOS demonstrated higher HOMA index levels of (2.77 ± 1.80 vs. 0.81 ± 0.08; p < 0.000) when compared with the control subjects. Furthermore, patients with PCOS showed an increased mean CIMT (0.63 ± 0.16 mm) when compared with the control subjects (0.33 ± 0.06 mm). This difference was statistically significant (p = 0.001).

The mean ± SD of brachial artery diameter at baseline was 3.89 ± 0.19 mm in normal subjects and 3.86 ± 0.11 mm in the PCOS group. The difference was not statistically significant (p = 0.19). Moreover, the mean ± SD of brachial artery diameter post ischemia was 4.13 ± 0.17 mm in normal subjects and 4.23 ± 0.12 mm in the PCOS group. The difference was statistically significant (p = 0.01).

The mean FMD in young patients with PCOS was 10.07 ± 1.2% and 6.5 ± 2.06% in normal subjects. The difference was statistically significant (p = 0.001). On the other hand, there was no significant association between HOMA index and CIMT in PCOS patients (r = +0.13; p = 0.18). The HOMA index of insulin resistance had a significantly negative relation with FMD in PCOS patients (r = -0.3; p = 0.02).

## Discussion

The endothelium is considered the largest endocrine gland, and secretes many transmitters to maintain the homeostasis of the circulatory system [8]. FMD is a non-invasive US method currently recognised as a useful technique for the evaluation of endothelial function [8]. The basic mechanism of FMD is the evaluation of brachial artery dilatation by evoking brachial artery ischemia. After brachial artery occlusion, endothelial nitric oxide is released and vascular smooth muscle relaxation occurs [9].

One of the early processes in the pathophysiology of atherosclerosis is impaired endothelial function [10]. Impaired endothelial function which is quantified by FMD is a marker of increased cardiovascular risk because it is well correlated with impaired endothelial function in coronary arteries [11]. The exact effect of PCOS on endothelial function remains controversial. Several studies have revealed that it is not impaired in women with PCOS who are either not obese or do not display morbid obesity [7,12,13]. However, some authors believe that endothelial function is impaired in patients with PCOS [5,14,15]. In our investigation, we evaluated vascular function in subjects with PCOS, and compared those patients with healthy control subjects.

Our study demonstrates a significant difference in CIMT between both age-matched PCOS and control subjects. Our result is in agreement with the report by Lukhani [16] and Talbott et al. [17] but is in contrast with the study of the Meyer et al. [4]. Our study demonstrates a significant difference in FMD between both PCOS and control groups, which is in agreement [9,18] and in contrast [7,19-22] with other studies. Orio et al showed that a significant difference in flow-mediated dilation and in intima-media thickness in young, normal-weight, nondyslipidemic, nonhypertensive women with PCOS in comparison with control subjects [14]. Although they excluded patients with dyslipidemia and hypertension from study group but our results are in concordance to the report by them.

Our results provide additional evidence confirming that there is endothelial dysfunction in women with PCOS in comparison with normal subjects.

The pathophysiological mechanism of inducing endothelial dysfunction remains unclear, but insulin resistance seems to be essential. Beckman et al. [7] reported an association between insulin resistance and endothelial dysfunction in type 2 DM and lipodystrophic diabetes. Others have shown an association between insulin resistance in children with DM and MS [23,24].

The postulated mechanisms whereby insulin resistance can adversely affect the endothelium are: overproduction of free fatty acids; tumour necrosis factor (TNF) α; leptin, which causes endothelial dysfunction; and the induction of an increased oxidative stress mechanism that, contributes to endothelial dysfunction [4,25,26].

In our study, there was a statistically significant difference in terms of BMI between PCOS and control subjects (both groups had normal BMI but the difference between them was statistically significant). Previous studies reported that the endothelial function was preserved in lean individuals and without morbid obesity with PCOS [8,13], but this still remains controversial [6,14,15]. We accept the lack of BMI matching as a limitation of our study, and recommend its consideration in future research.

Although in our study, the mean CIMT was different between PCOS and normal subjects, the HOMA index was correlated with FMD and we did not find a relationship between the HOMA index and CIMT. A previous study revealed that endothelial dysfunction occurred early in the development of atherosclerosis, preceding the onset of increased CIMT [27]. Thus, it seems that various risk factors in PCOS patients may contribute separately to the development of endothelial dysfunction.

We found that young patients with PCOS had higher levels of MS inclusion criteria such as serum TG, TC, LDL, FBS, insulin, insulin resistance (HOMA-IR) and lower levels of serum HDL. Thus, we believe it is better to consider PCOS as an ovarian manifestation of MS.

In conclusion, PCOS accompanies the tsunami of MS and hormonal abnormalities such as insulin resistance, dyslipidaemia, hyperandrogenaemia all make PCOS patients susceptible to future cardiovascular events. The diagnosis of this entity may offer an early cardio-protective protocol for women with PCOS.

## List of abbreviations

PCOS: Polycystic ovary syndrome; Met S: Metabolic syndrome; TG: Triglyceride; CIMT: carotid intima media thickness; TC: Total cholesterol; LDL: Low density lipoprotein; HDL: High density lipoprotein; IR: Insulin Resistance; FMD: Flow-mediated dilatation.

## Competing interests

The authors declare that they have no competing interests.

## Authors' contributions

All the authors in this manuscript have read and approve the final manuscript. MA: concept and design, and manuscript writing. AM: The Ultrasonographic studies and manuscript writing. MG: Data collection, Manuscript editing. LJ: Data Collection, concept and design. SS: Data analysis.
